# Supplementary material for: Time, talk, and teamwork: Perceptions of personalised dementia care planning conversations in primary care
Source: PLoS One. 2026 Sep 15;21(9):e0343482. doi: 10.1371/journal.pone.0343482 (PMC13577471; doi:10.1371/journal.pone.0343482)
Supplement: Supplementary file 1 [file pone.0343482.s001.docx]

# **Topic guide for people with dementia and carers**

**Introduction**

- ***Introduction to the interview:***

In this interview I would like to ask you some questions about **how professionals talk to you about what you need to help you get on with your life.** As you know, the overall aim of the research is to find out what training is needed for staff to be able to have these sorts of conversations with people with dementia and their chosen family/friends. We want to use what we learn in these interviews to develop training and guidance for staff.

***Run through the following with participants:***

- Remind participants that the interview will be audio recorded
- Informally observe for changes in capacity and check that the person still consents to their participation.
- If there are any doubts about capacity to consent, follow consent procedures set out in the protocol.
- Explain that the participant may decline to answer any questions, may take a break at any point or ask for the interview to be stopped completely.
- Explain that the participant can request that their data is withdrawn from the study at any point today or during the next six weeks.
- Reassure that there are no wrong answers

## **Guide questions**

1. **Care plans**

- Recap on the Participant Information Sheet e.g., National Health Service (NHS) England, who are the leaders of the NHS in England, say that everyone with dementia should have a care plan. National guidelines on dementia also recommend that everyone with dementia should have a care and support plan. The plan should say what the person’s unique needs and wishes are and how they will be addressed. It should include their carer’s needs as well where appropriate. The care plan should be updated every year. What is your experience of having a care plan (if any)?
- What do you think about the idea of a care plan?
- [if has experience of having a care plan] – can you tell me more about how the care plan was created? *(****Prompts****: who, how, were things agreed with you? Was it shared with you? What format was it in e.g., hard copy, emailed to you?)*
- What things do you think professionals should ask you about to be able to create a care plan that helps you to get on with your life? *(****Prompts:*** *Physical health, medications, mental health, relationships with family/friends/others, hobbies, engagement with local community groups (church etc), finances, planning ahead, Power of Attorney….)*

2. **Experiences of care planning conversations**

- Have you had conversations with health or social care professionals to find out what matters to you and what you need to live your life? Could tell you tell me about that/those conversations?
- [If interviewee talks about specific conversation/s] – **Probe:** Can you tell me more about that conversation? What was good/not so good about it? Do you have other good/bad experiences of conversations with professionals you could tell me about? What was it that was good/not so good? *(****Prompts:*** *Types of questions, how you were addressed, timing/duration, non-verbal aspects such as eye contact, positioning, multi-tasking, use of props/showing you visual resources like pictures, maps, calendars, things written down….etc)*

### Follow up question

- Did things change as a result of the conversation? If so, what changed? *(****Prompts:*** *were you aware of a care plan being created? If so, what did it cover? Were any changes made as a result?)*
- How would you want professionals to have a chat with you and your chosen family member/friend about what is important to you, to help you live your life (***Prompts:*** *Think about the sorts of questions should they ask, how they should address you, timing/duration, ways in which things should be said, how you prefer to be asked about your background in terms of beliefs, ethnicity, the way you live your life, the things you like to do, sexuality…, what feels like a safe space, balancing contributions of the person with dementia and carer, what to think about if conversations are remote and/or involvement of an interpreter – e.g., were you able to have the conversation in the language you wanted to?)*
- In the conversations you have had with professionals, what’s missing? What would you like them to ask about that could help you in your life?
- What topics might be difficult to talk about and how should professionals approach/not approach these e.g., what questions should they ask?
- What training do professionals need to have better conversations with people with dementia and their carers?

## **Feedback on research activities**

- How have you found answering the questions today?
- Is there anything you would like to tell us about your experience of taking part in the study?
- Is there anything we could have done to make it easier for you to take part?

## **Wrapping up**

- Is there anything else you would like to tell me about how professionals can have better conversations with people with dementia and carers about what they need to be able to get on with their lives?

If the person had unanswered questions about the dementia, explore when and how they could raise these; check whether they would like you to put them in touch with an organisation who should be able to help (e.g., Alzheimer’s Society; GP surgery)

Thanks, any questions for us, reminder re confidentiality.

# **Topic guide for practitioners**

**Introduction**

- ***Provide introduction to the interview:***

In this interview I would like to ask you some questions about **how professionals talk to people with dementia and carers about their care and support needs.** As you know, the overall aim of the research is to find out what training is needed to help staff to have these sorts of conversations with people who have dementia and their chosen family/friends. We want to use this information to develop training and guidance for staff.

***Run through the following with participants:***

- Remind participants that the interview will be audio recorded
- Check that the practitioner still consents to their participation.
- Explain that the participant may decline to answer any questions, may take a break at any point or ask for the interview to be stopped completely.
- Explain that the participant can request that their data is withdrawn from the study at any point today or during the next six weeks.
- Reassure that there are no wrong answers

## **Guide questions**

1. ***Care Plans***

- National Health Service (NHS) England, who are the leaders of the NHS in England, say that everyone with dementia should have a care plan. National Institute for Health and Care Excellence (NICE) guidelines on dementia also recommend that everyone with dementia should have a care and support plan. Have you been involved in creating care plans with people with dementia and their carers? What has that been like for you?
- What areas of care and support would you cover with a person with dementia and/or their carer? *(****Prompts:*** *Physical health, mental health, relationships with family/friends/others, hobbies, engagement with local community groups (church etc), finances, planning ahead, Power of Attorney….)*
- Where do care planning conversations normally take place?
- How long do you meet people for?
- What do you think are the challenges of creating dementia care plans?
- What format do you think a care plan should take? *(Prompts: paper/electronic form, what sections should it include?)*

2. ***Team working***

- What are your experiences (if any) of working with other practitioners to develop care plans for people with dementia? (it doesn’t matter if you haven’t had this experience).
- What types of practitioners, in your experience, are involved in dementia care planning?
- Where do roles overlap? (e.g., DA and SPLW)
- What are the challenges of this collaborative approach to care planning (e.g., how well do practitioners work together)? What causes these challenges? *(****Prompts:*** *think about how things normally operate within your organisation/relationships within and outside of the organisation)*
- What ideas do you have about how the challenges could be overcome?
- Tell me about example/s of good collaboration in dementia care planning. What was it that made it good?
- What is your experience of care plans being shared amongst the team and with the person with dementia and carer? What are the challenges of this? How might they be overcome? *(****Prompt:*** *What systems need to be in place to make it work?)*

3. ***Communication with people with dementia and carers***

Many people with dementia have communication difficulties. They may find it difficult to understand what people are saying, especially when the language used is complex and the conversation moves at a fast pace. They may lose their train of thought, forget words, say the wrong words, or be generally less verbal than they used to be. Some people may also have significant hearing or visual problems, making the communication problems even more challenging.

- Tell me about how you try to get around these communication problems. What do you do that seemed to help/not help? *(****Prompts:*** *think about types of questions, the way in which things were said, non-verbal communication, use of visual resources e.g., aspects of the environment/use of props, written words, objects, pictures…?)* **Probes:** What did this lead to? What happened? What was the outcome?)
- What do you find difficult about having these conversations with people with dementia?
- Have you been in a situation (in person or remote) where the carer seems to be answering all the questions? What did you do that seemed to help/not help? **Probes:** What did this lead to? What happened? What was the outcome?
- It can be hard to make sure a care planning conversation covers both the needs of the person with dementia and those of the carer. How would you normally try to find out both these things? *(****Prompts:*** *Would you ask things in a certain way? Would you see them separately?)*
- Do you have examples you’d like to tell me about where communication was challenging in these care planning conversations? Tell me what happened. **Probes:** what things did you try? What seemed helpful/not helpful?
- What topics are harder than others to bring into the conversation? IWhat makes those topics difficult? Have you developed ways around that?
- What would you do or say to try and understand a person with dementia and carer’s unique cultural needs? *(****Prompts:*** *what types of questions would you ask? What resources might you use?)*
- Often people with dementia have other long term health conditions. How do you think care planning conversations should address this?
- Do you have suggestions that we haven’t already talked about, for ways professionals can communicate with people with dementia and carers to best understand their care and support needs?
- As you know, the aim of this study is to develop training and guidance to support practitioners to have care planning conversations with people with dementia and carers. What support and guidance would you like?
- Do you have suggestions for what a training and guidance toolkit should look like (e.g., what format would be most helpful)? *(****Prompts:*** *written guidance, videos, infographic…other?)*
- What do we need to think about when putting this sort of training and guidance into practice? E.g., What might the challenges be? *(****Prompts****: current systems of everyday working/the culture of the organisation/capacity to implement it/practicalities/relationships, partnerships, connections).* How might those challenges be overcome?

## ***Feedback on research activities***

- How have you found answering the questions today?
- Is there anything you would like to tell us about your experience of taking part in the study?
- Is there anything we could have done to make it easier for you to take part?

## ***Wrapping up***

- Is there anything else you would like to tell me that we haven’t covered?
- [If linked to recruiting site for observations] Would you be happy to be invited to take part in an observation (explain what this entails)?
- Would you be happy to be invited to test out the training and guidance toolkit that we develop as a result of this research (explain what this entails)?

Thanks, any questions for us, reminder re confidentiality.
